# Glioblastoma Stem Cells Microenvironment: The Paracrine Roles of the Niche in Drug and Radioresistance

**DOI:** 10.1155/2016/6809105

**Published:** 2016-01-06

**Authors:** Alessia Fidoamore, Loredana Cristiano, Andrea Antonosante, Michele d'Angelo, Erica Di Giacomo, Carlo Astarita, Antonio Giordano, Rodolfo Ippoliti, Elisabetta Benedetti, Annamaria Cimini

**Affiliations:** ^1^Department of Life, Health and Environmental Sciences, University of L'Aquila, 67100 L'Aquila, Italy; ^2^Sbarro Institute for Cancer Research and Molecular Medicine, Temple University, Philadelphia, PA 19122, USA; ^3^Department of Medicine, Surgery and Neuroscience, University of Siena, 53011 Siena, Italy; ^4^National Institute for Nuclear Physics (INFN), Gran Sasso National Laboratory (LNGS), Assergi, 67100 L'Aquila, Italy

## Abstract

Among all solid tumors, the high-grade glioma appears to be the most vascularized one. In fact, “microvascular hyperplasia” is a hallmark of GBM. An altered vascular network determines irregular blood flow, so that tumor cells spread rapidly beyond the diffusion distance of oxygen in the tissue, with the consequent formation of hypoxic or anoxic areas, where the bulk of glioblastoma stem cells (GSCs) reside. The response to this event is the induction of angiogenesis, a process mediated by hypoxia inducible factors. However, this new capillary network is not efficient in maintaining a proper oxygen supply to the tumor mass, thereby causing an oxygen gradient within the neoplastic zone. This microenvironment helps GSCs to remain in a “quiescent” state preserving their potential to proliferate and differentiate, thus protecting them by the effects of chemo- and radiotherapy. Recent evidences suggest that responses of glioblastoma to standard therapies are determined by the microenvironment of the niche, where the GSCs reside, allowing a variety of mechanisms that contribute to the chemo- and radioresistance, by preserving GSCs. It is, therefore, crucial to investigate the components/factors of the niche in order to formulate new adjuvant therapies rendering more efficiently the gold standard therapies for this neoplasm.

## 1. Introduction

Cancer stem cells (CSCs) were first isolated in acute myeloid leukemia (AML) patients proving that CSCs are able to reproduce many features of human AML in immunodeficient mice [1]. The presence of CSCs has been then reported in a series of solid tumors including breast, lung, prostate, colon, and brain tumors [2–7]. The brain has been for a long time defined as an organ with limited regeneration ability, until the discovery of neural stem cells in adult brain [8–10]. It is now known that populations of stem and progenitor cells located in distinct regions of the mature brain ensure the continued neurogenesis process in adults. Similar cells with the capacity of self-renewal are identified in other tissues. These cells are undifferentiated and mitotically active; thus, they may potentially give rise to cell transformation into tumor stem cells [11]. The presence of cells with stem-like properties in human brain tumors was firstly demonstrated by Ignatova et al. [12], who isolated clonogenic, neurosphere-forming precursors from postsurgery specimens of human glioblastoma and medulloblastoma [12]. Following this finding, many studies reported the existence of neurosphere-forming cells in various grades of gliomas [6, 13–19].

*In vitro*, stem-like cells derived from brain tumors have high regenerative capacity and the ability to differentiate into neuronal or glial cells as they can express specific neural markers. Furthermore,* in vivo*, cells from glioblastoma (GBM) neurospheres were reported to induce tumorigenesis, even in serial transplantation settings. Brain tumor stem cells* in vitro* showed many stem-cell features such as extensive self-renewal, multipotency, and generation of many progenies. The tumors developed in mice model injected with glioblastoma stem cells (GSCs) display high extensive migratory and infiltrative capacity, indicating that isolated brain tumor stem cells* in vivo* may induce tumor to the brain similar to those observed in glioblastoma multiforme [7, 14, 15].

Many scientific reports still debate on the origin of brain tumors, particularly whether they may derive from the dedifferentiation of a brain cell or from the transformation of a neural stem cell (NSC) or progenitor cell [20]. Several hypotheses have been proposed about the nature of the neural cell type that is the target of the transformation resulting in tumorigenesis (Table 1) [21–34]. Several reports indicate that brain tumors might rise from the transformation of undifferentiated precursor cells, which are located not only in germinal regions of the developing and early-postnatal CNS, but also in regions of mature brain in which neurogenesis persists throughout adulthood [11]. There are two identified neurogenic niches in the adult mammalian brain: the subventricular zone (SVZ) of the forebrain lateral ventricles and the subgranular zone (SGZ), in the dentate gyrus of the hippocampus, in which both quiescent stem cells and mitotically active progenitor cells reside [35]. It was suggested that SVZ represents the most likely site of origin of gliomas [36], although the site of tumor development is often different from the site of origin of glioma: in fact, a brain tumor stem cell, through asymmetric divisions, might generate another brain tumor stem cell, remaining within the SVZ, and also a progenitor cell that migrates away to form the tumor mass. When a differentiated cell accumulates mutations on oncogenes, it may undergo a dedifferentiation process and give rise to brain tumors. In the same way, a NSC, with a long lifespan, capable of self-renewal can easily accumulate mutations and gives rise to a cancer cell [11]. Furthermore, it is worth noting that many researches support the hypothesis that it is the deregulation of specific genetic pathways, rather than cell of origin, that determines the appearance of the phenotype of high-grade gliomas, suggesting that glioma may originate from cells at any differentiation stage during glial development [20, 37]. Although, the cell type involved in the different genetic forms of glioma is still undefined [38], the resultant GSCs show neural stem cell (NCS) properties in terms of self-renewal capacity, multilineage differentiation potential, telomerase activity, expression of stemness markers, surface receptors and ABC transporter proteins, production of growth and angiogenic factors and cytokines, ability of motility-migration, and specific signaling pathways [7, 36, 39, 40]. Particularly the crucial role of the microenvironment in both NSCs and GSCs is just emerging. In the healthy brain, NSCs commonly are situated in perivascular regions, characterized by restricted oxygen availability and distinct extracellular cellular matrix profiles. In fact, it is demonstrated that these specialized niches support neurogenesis and regeneration after injury [41–43].

The regulation of stem cell division and fate is strongly dependent on specific anatomical elements and paracrine factors such as the cell-cell interactions, proximity to the cerebrospinal fluid of the lateral ventricle (in the case of SVZ), association with blood vessels, and extracelular matric (ECM) biology. The astrocytes and ependymal cells of SVZ regulate stem cell niche, as the former establishes close contacts with all cell type and with blood vessels, sensing and integrating any signals from germinal regions and vasculature within stem cell niche. The line the lateral ventricles from which take factors from the cerebrospinal fluid. Ependymal cells prevent glial differentiation of SVZ cells, since they produce noggin, an antagonist of BMP signaling, and in addition, along with SGZ precursors, they express CXCR4, the receptor for the chemokine stromal cell-derived factor-1 (SDF-1), which secreted by meninges and interacting with Sonic Hedgehog (SHH) regulate the cerebellar and hippocampal development. Several factors are produced in the niche that influence the germinal status such as EGF, bFGF, IGF1, TGF-*α*, VEGF, Eph/ephrin signaling, Shh, prolactin, and adrenal hormones. As the neurogenic niches are situated closely with blood vessels, the vascular system actively controls the neurogenic process. In fact, both the ncieh and the vessels are stimulated by the same factors including bFGF, VEGF, IGF-1, and TGF-*α* and endothelial cells produce mitogens and differentiation and survival factors of neurons (bFGF, IGF-1, VEGF, PDGF, IL8, and BDNF) [42]. In addition, SVZ may have a modified BBB, as stem cells and the transit-amplifying cells make direct contact with blood vessels, having access to molecules of the blood stream such as growth factors, hormones, nutrients, and oxygen. Finally, the complex of basal lamina and ECM (in SVZ) constitutes a site of integration of niche signals from the vasculature, including pericytes, endothelial cells (ECs), and factors from the blood, from ependymal cells, mesenchymal cells, axon terminal, and the cerebrospinal fluid [44]. It regulates proliferation and migration as it can increase ligand activity or hold ligands as bound store. It was shown that heparin sulfate proteoglycans bind several factors fundamental in adult neurogenesis, such as morphogens and mitogens (BMP-2–4, HH, and Wnts), components of the ECM (collagens, laminins, and tenascin), growth factors (EGFs, FGFs, IGF-II, PDGF-AA, and VEGF), chemokines, and cytokines [42]. The studies about the neural stem cell niche demonstrate how elaborate is the microenvironmental architecture and how sophisticated is the balance of niche components and factors involved in stem cell regulation. The GBM niche resembles even if in an aberrant way the precise network that governs the cancer stem cell proliferation, tumor progression, metastasis, and resistance to therapies.

In this review, we will analyze the phenotype and the components of the brain tumor niche and debate over the tight correlation between GSCs and their microenvironment and its impact on tumor biology and on the drug and radioresistance.

## 2. Glioblastoma Microenvironment and Resistance to Chemo- and Radiotherapy

### 2.1. Vascular and Hypoxic Phenotype of Glioblastoma

Among all solid tumors, the high-grade glioma appears to be the most vascularized one. In fact, vascular proliferation known as “microvascular hyperplasia” is a hallmark of GBM, characterized by rapidly dividing endothelial cells that form microaggregates of sprouting vessels and of smooth muscle cells/pericyte known as “glomeruloid bodies” [45]. The process of tumoral angiogenesis is a complex series of events induced by the interaction between cells and extracellular environment. Capillaries in tumors form a complex network with different features compared to that of normal tissues. The main differences reside in structural and functional abnormalities such as dilatations, incomplete or absent basement membranes, high permeability, irregular architecture, blind ends, absence of vascular smooth muscle, and pharmacological/physiological receptors [46, 47]. Since all these altered characteristics in the vasculature determine irregular blood flow and tumor cells spread rapidly beyond the diffusion distance of oxygen (about 100 *μ*m) in the tissue, O_2_ as well as nutrients is supplied with increasing difficulty and hypoxic or anoxic areas develop throughout the tumor mass [47]. The immediate cellular reaction to this event is the induction of angiogenesis with the consequent formation of new vessels to supply oxygen to the tumor cells, a process mediated by hypoxia inducible factors (HIFs). However, this new capillary system is not efficient in maintaining a proper oxygen supply to the growing tumor mass, thereby causing an O_2_ gradient within the neoplastic zone, a feature that is present in all solid tumors [47]. Although in healthy brain tissue the physiological oxygen concentrations range between 12.5 and 2.5% (pO_2_ = 200 to 100 mmHg), the majority of GBM presents mild to moderate/severe hypoxia, with oxygen concentrations ranging between 2.5 and 0.5% (pO_2_ = 20 to 4 mmHg) for mild hypoxia and 0.5 and 0.1% (pO_2_ = 4 to 0.75 mmHg) for moderate/severe hypoxia [48–50]. Necrotic areas within the tumor mass, which represents another hallmark of GBM, are commonly characterized by severe hypoxia and cells within the tumor may survive due to molecular or genetic changes as the result of the inadequate supply of O_2_ and nutrients [51, 52]. Expanding neoplastic cells are often found by microscopic examination of solid tumors close to blood vessels and in the core of necrotic areas [53]. These necrotic regions are linked spatially and temporally with the microvascular hyperplasia and are characterized by the presence of pseudopalisading necrotic areas in which neoplastic astrocytes are located around necrotic centers. Tumor necrosis may arise from increased apoptosis or increased growth beyond the capacity of the emerging blood supply [45]. All GBM tumors have intratumoral necrosis to a varying degree but it does not seem to be related to tumor size, as it is found in both small and large tumors [52].

Absent/low intratumoral oxygen constitutes a serious problem for the treatment of glioma by radiotherapy, as tumor cells resulted radioresistant when the pO_2_ pressure within the tumor is low. In fact, in hypoxia a radiation dose three times higher than in the presence of normal oxygenation is needed, and the DNA damage, induced by formation of free oxygen radicals, appeared reduced and can be repaired more easily [54, 55]. In addition, after radiation and in hypoxic condition high amounts of nitric oxide (NO) and superoxide (O_2_^−^) radicals are detected in the tissue, which are involved in several aspects of tumor development and progression [56, 57]. Moreover, NO can react with superoxide forming the highly reactive compound peroxynitrite (ONOO^−^) [58], which reacts with tyrosine residues of target proteins (nitration) such as p53, leading to its functional inactivity and to glioma progression [59, 60]. It was demonstrated that radiation-induced NO radicals are involved in the induction of radioresistance* in vitro* [56]. These mechanisms reveal that the low level of oxygen strongly impacts on the reduction of the apoptotic potential of tumor cells, thus promoting radioresistance. Moreover the poor blood perfusion and the fluctuating oxygen state increase glycolytic pathway and acid production by the upregulation of the Warburg effect, resulting in a tumor pH highly acidic [61, 62]. Electrode measurements of pH in human brain tumors detected low level of pH 5.9 with a mean around 6.8, whereas normal brain tissue has a pH of 7.1 [45]. The acidic stress is an important component of glioma microenvironment and in many tumors could affect several biological processes, including proliferation, angiogenesis, immunosuppression, invasion, and chemoresistance [62–65]. Reduced pH may increase the resistance of glioma cells to multiple drugs including topotecan and cisplatin, although it decreases cell growth [66, 67] and may also influence the cytotoxicity of anticancer drugs, inhibiting both their diffusion across the membrane and their active transport. In fact many molecules, which diffuse passively across the cell membrane most efficiently in the uncharged form, at low pH result protonated and display decreased cellular uptake [68]. Many solid tumors show altered lymph vessels compared to normal tissues inducing an increase of interstitial fluid pressure that prevent the distribution of larger molecules and constrict the blood vessels so that blood flow is diverted away from the center of the tumor toward the periphery [69]. The altered vascular and lymphatic system and the organization of the extracellular matrix within the tumor mass may compromise an effective drug delivery and penetration toward the target tumoral site [68].

### 2.2. Hypoxic/Perivascular Niches

**Figure 1:**
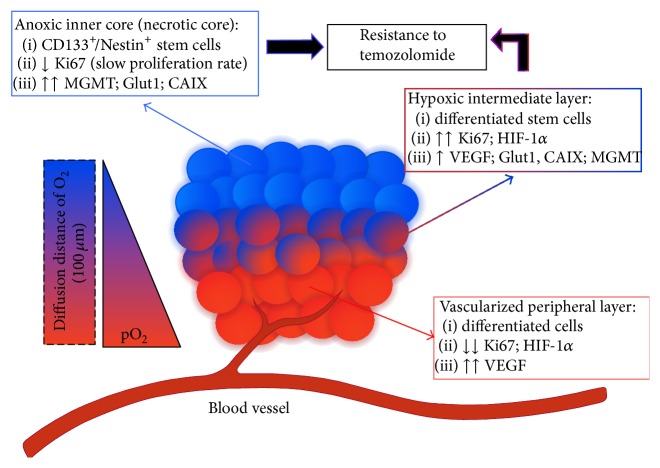
pO_2_ gradient model for glioblastoma niche.

In order to understand the mechanisms belonging to the tumor microenvironment that influence the responses of GSCs to chemo- and radiotherapy, it is important to describe the features of the anatomical locations known as niches in which they reside. The tumoral cytoarchitecture of GBM consists of “normoxic cells” mostly located in the periphery of the tumor mass and most of hypoxic cells situated in the center and necrotic/dead cells in the inner cores as a response to the O_2_ gradient. GSCs may therefore be located in distinct niches, differing in their tumor-initiating capacity, expression of markers, and susceptibility to therapy (Figure 1) [70, 71]. Particularly GSCs are highly enriched in both vascular and necrotic/hypoxic niches [72] which not only are an anatomical and structural unit where stem cells reside, but also may be a functional and specialized microenvironment which, through complex and dynamic pathways, supports their expansion and spread [71, 73, 74] and furthermore regulates their cell-renewal and fate [36]. This microenvironment maintains cells in a “quiescent” state preserving their potential to proliferate and differentiate, thus protecting them by the effects of chemo- and radiotherapy [36]. In the brain, the development of multiple vascular GSC niches may be one of the main factors promoting brain tumor growth and invasion [75]. The vascular niche may function to chemoattract tumor cells, promote their transition to a “stem-like” phenotype, and support their maintenance and proliferation. This dynamic system is the result of the interaction between tumor cells, endothelial cells, pericytes, and tissue specific components, for the maintenance of the tumor stem cell population [76]. Therefore, direct genetic alterations or deregulated crosstalk between signaling pathways of the GSCs and the cells of their niche may assume the role of important determinants of functional tumor microenvironment preceding cancer development [36]. The localization of GSCs in close proximity to blood vessels, in perivascular niches, is crucial, as GSCs can establish a bidirectional and supportive interaction with vascular system, especially with endothelial cells, through several mechanisms that include coopting preexisting vessels and inducing angiogenesis, in order to ensure their maintenance [77] In fact, it was demonstrated that CD133^+^ GSCs may produce vascular endothelial growth factor (VEGF), promoting endothelial cell growth, migration and formation of vascular tubular structure in culture, and so their tumor initiating capacity. The inhibition of VEGF axis through the treatment with bevacizumab shows paracrine effects on endothelial cells and other cancer cells thus reducing tumor growth in mice injected with CD133^+^ GSCs in terms of weight, vascularity, and hemorrhage [78]. In addition, endothelial cells can interact specifically with Nestin^+^/CD133^+^ cancer cells, located in proximity of capillaries, and participate in the maintenance of their self-renewing and undifferentiated state, supplying with secreted factors. Moreover, these cells, when cotransplanted with the Nestin^+^/CD133^+^ cancer cells, promote and accelerate the initiation and growth of orthotopic brain tumor xenografts [75]. In both* in vitro* and* in vivo* studies, it was found that endothelial cells, when cocultured with glioma cells, promoted the phenotype of CSC-like glioma cells, increasing the expression genes such as Sox2, Olig2, Bmi1, and CD133 and their tumorigenicity. In fact, endothelial cells in the tumor microenvironment induce GSCs properties and tumor propagation by activating the Hedgehog signaling pathway in glioma cells [79]. Interestingly, the GSCs may contribute directly to tumor vasculogenesis, as they can transdifferentiate into endothelial cells. In fact, it was found that a significant part of endothelial cells in GBM has neoplastic origin since they share the genetic alterations with tumor cells. The culture of GSCs in endothelial conditions produced endothelial cells and orthotopic or subcutaneous injection of GSCs in immunocompromised mice generated tumor xenograft, with a vasculature composed of human endothelial cells [80, 81]. The incidence of the transdifferentiation phenomenon in GBM specimens and its involvement in the structure and function of tumor niche is still to be studied. In addition to regulating stem cells proliferation and fate, niches play a protective role, defending stem cells from environmental/exogenous insults. Thus, niches may be able to protect CSCs from chemo- and radiotherapies, allowing these cells to give rise to a new tumor mass following an initial clinical response. Particularly endothelial cells play a central role in the protection of stem cells and tumor cells from radiation damage, evidence reports that solid tumor xenografts grown in mice with radiation-resistant endothelial cells are much less susceptible to radiation damage than tumors grown in wild-type mice [82]. Moreover, the bidirectional interaction between endothelial and tumor cells may regulate radiation responses. It was demonstrated that the coculture of glioma and endothelial cells in a 3-dimensional system determines a survival advantage after irradiation for this type of vascular system compared to blood vessels constituted of endothelial cells alone. Similarly, monoculture of endothelial cells, after radiation, showed higher level of apoptosis than when they were cocultured with glioma cells [83]. Targeting the perivascular niche, through antiangiogenic therapies, represents a promising approach to prevent tumor progression, but the adoption of VEGF antagonism (bevacizumab) is insufficient to inhibit the formation of new GBM stem cell niche, as it seems to increase the expression of proangiogenic factors as FGF1 and FGF2 and CXCL12 [84, 85] and the recruitment of proangiogenic bone-marrow derived cells [86]. In addition, the ability of GSCs to transdifferentiate into endothelial-like cells may give rise to an alternative microvasculature through a VEGF-independent angiogenic process [80, 87]. Finally, antiangiogenic treatments of GBM may produce a shift from angiogenic to infiltrative phenotype, leading to the development of more hypoxic microenvironment [88, 89].

## 3. Paracrine Factors of the Niche

**Figure 2:**
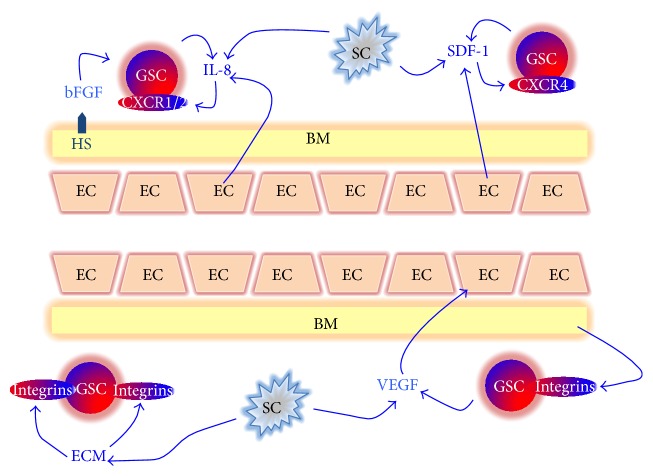
Schematic representation of interactions between glioma stem cells and components of microenvironment. Blue arrows indicate a positive regulation in terms of proliferation and/or radioresistance. EC, endothelial cells. SC, stromal cells. GSC, glioma stem cells. bFGF, basic Fibroblast Growth Factor. BM, basement membrane. ECM, extracellular matrix. HS, Heparin Sulphate. IL-8, interleukin-8. SDF-1, stromal cell-derived factor-1.

A plethora of soluble and cell-surface molecules have been identified within the vascular niche, which through paracrine and/or autocrine mechanisms have been demonstrated to regulate self-renewal and angiogenesis in GBM (Figure 2).

### 3.1. KIT Ligand and PEDF

KIT ligand (also known as stem cell factor), a potent glioma-derived proangiogenic factor [90], promotes the migration, survival, and proliferation of neural progenitor cells [91, 92]. Different studies demonstrated that pigment epithelium-derived factor (PEDF), a niche derived regulator of NSCs, maintains their self-renewal. Ramírez-Castillejo et al. showed that PEDF is secreted by components of murine SVZ and stimulates self-renewal in adult NCSs* in vitro* and its intraventricular infusion promotes the cycling of slow-dividing stem cells [93, 94]. PEDF may cooperate with Notch to regulate stemness in the vascular niche, activating the Notch signaling-dependent self-renewal in adult periventricular NSCs [95]. PEDF has multiple biological properties, not only neurotrophic, but also neuroprotective, antitumorigenic, and potent antiangiogenic activity [96]. Because of its antiangiogenic activity, recent studies have shown that decreased PEDF expression is associated with a higher intratumoral microvessel density and a more metastatic phenotype in several tumors, such as prostate and hepatic carcinoma, gliomas and lymphangiomas [97–100]. Its low expression has been correlated with the increased incidence of metastasis and poorer prognosis [97–99, 101]. Despite its antitumorigenic action, it was demonstrated that PEDF, as autocrine factor, is secreted by GSCs and stimulates self-renewal activity, controlling stemness and tumor progression. The activation of EGFRvIII/STAT3/PEDF signaling regulates the self-renewal of infiltrative GSCs, as EGFRvIII+/PEDF_high_ GSCs resulted to be responsible for glioma infiltration. In addition, PEDF maintains glioma stemness and self-renewal ability by activating Notch/Sox2 signaling axis and its silencing reduces the infiltration of GSCs and increases the survival of tumor bearing mice. As the levels of PEDF are correlated from grade II glioma to grade IV GBM, PEDF may be an indicator of infiltrative GSCs and a prognostic marker of low grade glioma [102].

### 3.2. CXCL12/SDF-1

The stromal cell-derived factor-1 (SDF-1) or C-X-C motif ligand 12 (CXCL12) controls the normal stem/progenitor-cell trafficking and homing in the central nervous system and maintains stem cells in the neural niche [103]. CXCL12 is the only ligand for CXCR4 (C-X-C motif receptor 4) and acts as autocrine/paracrine growth factor for several cancers [104–108], including GB [109–111]. CXCR4 shares its ligand with CXCR7 [112, 113] which may be a role in tumorigenesis [114]. Within the tumor tissue, distinct stromal cells express CXCL12 and it may be mainly secreted by stromal fibroblasts in tumor tissue [115]. The activation of CXCL12 pathways may be involved in tumor progression and in the resistance to the conventional therapies both directly, promoting cancer cell proliferation/survival, invasion and cancer stem, and/or tumor-initiating cell phenotype, or indirectly, recruiting stromal cells to favor tumor relapse, metastasis, and angiogenesis [116–119]. In fact, CXCL12 can activate phosphoinositide 3-kinase (PI3K)/Akt, IP3, and mitogen activated protein kinase (MAPK) pathways via CXCR4 [112, 120, 121] and PLC/MAPK pathway via CXCR7, increasing cell survival in gliomas [122]. Moreover, CXCL12 is responsible for the recruitment of several BMDCs expressing CXCR4, including myelomonocytes, endothelial precursor cells, and “hemangiocytes” that may directly determine neorevascularization [123–125]. Glioma cancer cells express both CXCL12 and its receptors. The CXCR4/CXCL12 axis is particularly active in pseudopalisading areas surrounding the necrotic foci and in invading glioma cells [126, 127]. It was shown that CXCR7 was found on “differentiated” glioma cells, which mediated their resistance to apoptosis, whereas CXCR4 is expressed in GSCs [122]. In fact, it was suggested that CXCL12 may be one of the regulators of the biological activity of CSC [116, 117]. CXCL12 produced by brain endothelial cells chemoattracts and sustains proliferation of primary human GBM cells [128]. In fact, it was demonstrated that exogenous CXCL12 can induce glioma cell proliferation and that CXCL12-dependent initiation of ERK1/2 and AKT pathways is involved in the transduction of proliferative signals in normal and tumor glial cells [110, 129]. CXCL12 can also control tumor cell apoptosis, activating NF-*κ*B [130], which represses radiation-induced tumor necrosis factor *α* (TNF*α*) production and tumor apoptosis [131]. In addition, CXCL12 can protect tumor cells from apoptosis induced by chemotherapeutic drugs activating antiapoptotic pathways and modulating the attachment of cancer cells through the regulation of integrins [132, 133]. Several reports showed that the treatment with VEGFR inhibitor determined the activation of CXCL12/CXCR4 pathway, increasing circulating CXCL12 levels and CXCR4^+^ cells and the infiltration of myeloid BMDC in brain tumors [85, 134] and promoting angiogenesis, tumor growth, and metastasis [135]. In addition, the treatment with chemotherapeutics or vascular-disrupting agents and irradiation exerts the same effects on circulating CXCL12 levels and on the recruitment of BMDCs [86, 136–138]. However, in contrast to monotherapy, the association of anti-CXCL12 therapy with other anticancer therapies could be more efficacy since, as we have described above, therapeutic treatments activate the CXCL12 pathway that contribute to mechanisms of resistance to them according to the results from recent preclinical and clinical studies of antiangiogenic therapy, chemotherapy, or radiation therapy [139].

### 3.3. VEGF

The mammalian VEGF family of ligands consists of five glycoproteins referred to as VEGF-A, VEGF-B, VEGF-C, VEGF-D, and placenta growth factor (PlGF). The VEGF ligands bind to and activate three structurally similar type III receptor tyrosine kinases, VEGFR-1, VEGFR-2, and VEGFR-3. The VEGF family of ligands has distinctive binding specificities for each of these tyrosine kinase receptors, which contribute to their diversity of function. In response to ligand binding, the VEGFR tyrosine kinases activate several signaling pathways [140]. The tumoral “angiogenic switch” is driven by several proangiogenic factors in malignant glioma, among which VEGF and several additional, biologically active VEGF variants are produced by tumor cells, infiltrating inflammatory cells, and platelets and can be sequestered in the extracellular matrix. VEGF expression in malignant gliomas is most localized to areas of necrosis and hypoxia, including cellular pseudopalisades at the tumor leading edge. High levels of VEGF predict glioma aggressiveness and poorer outcome. Several hypoxia-dependent and hypoxia-independent mechanisms regulate the production of VEGF in the microenvironment of malignant gliomas. Hypoxia, via HIF-1*α*, activates VEGF and VEGFRs. The activity of VEGF is also increased by the aberrant activation of multiple growth factor receptors in malignant glioma, including EGFR platelet-derived growth factor receptor (PDGFR), scatter factor/hepatocyte growth factor receptor (MET), IGF receptor (IGFR), stem cell factor receptor (c-Kit), and FGF receptor (FGFR), and by the deregulation of signaling pathway such as phosphatidylinositol 3-kinase (PI3K/Akt) and Ras/MAPK pathways [141]. Several studies report that although VEGF is the main angiogenic factor in tumor, the therapy based on VEGF targeted agents may not be sufficient to inhibit tumor regrowth. This may be explained by the uprising of compensatory mechanisms for tumor angiogenesis that determine resistance to this therapy. An increased expression of angiogenic factors, after the anti-VEGF treatments, such as PIGF and an activation of the notch ligand/receptor system, which leads to the formation of a more mature tumor vasculature network, was found. Recent investigation has found a specific myeloid cell population that in response to the granulcyte colony-stimulating factor (G-CSF), IL-6, and CXCL12 factors, secreted by tumor cells and stroma, is mobilized and migrates to tumor site, thus mediating tumor angiogenesis and resistance to anti-VEGF therapy [140]. In addition, it was observed that culture of glioma cell lines in medium containing high levels of radiation-induced VEGF enhances the activation of VEGFR2 pathway and cell motility [142].

### 3.4. IL-8

Numerous chemokines, particularly interleukin-8 (IL-8), have emerged as promigratory and proangiogenic stimuli in multiple cancers and as regulators of GSCs functions [143]. IL8 effects are mediated by binding to either of two related G protein coupled receptors, CXCR1 and CXCR2, whose expression differs by cell type and throughout pathogenesis [143]. IL-8 can furthermore mediate invasion of the bulk of glioma cells and has been correlated with increased tumor grade in astrocytic neoplasms [144–146]. In addition, this chemokine drives the GBM tumor vascularization increasing the apoptotic resistance of ECs and inducing the expression of matrix-remodeling enzymes involved in endothelial sprouting [144, 147]. Although it has been recently revealed that autocrine IL-8 signaling contributes to GSCs self-renewal within tumors such as breast and liver [148, 149], its impacts on the behavior of GSCs, signaling with ECs of the perivascular niche, and participation in GBM tumor growth have yet to be elucidated. Recent findings show that IL-8 functions as a critical mediator supporting GSCs growth and migration toward ECs, a possible explanation of their perivascular colocalization in the GBM tumor microenvironment [150]. It was demonstrated that the treatment of breast cancer with chemotherapeutic drugs induces cellular apoptosis in the differentiated tumor cells, with the consequent FAS-mediated bystander effect and the concomitant production of Il-8 from the injured cells. The activation of the pathway Il-8/CXCR1 stimulates breast cancer stem cells and protects them from apoptosis. This phenomenon promotes the increase of CSCs after chemotherapy and tumor recurrence [148]. In addition, the production of Il-8 induced by anticancer drugs increases the expression of ABC transporter and side population in human hepatocellular carcinoma [151]. Further studies, however, are needed to elucidate similar roles of IL-8 in GSCs resistance to therapy.

### 3.5. Oxygen Gradient and Hypoxia

The niche and the oxygen tension gradient within the tumor mass play a crucial paracrine role in the definition of cell phenotype. Pistollato et al. [152] made a correlation between the GBM phenotype and the hypoxic gradient, giving a definition of a tumor stem cell concentric model niche. They found that it is possible to assign three concentric layers each containing diverse cell phenotypes analyzing the peripheral layer, the intermediate area, and the central core of nine GBM biopsies. The peripheral layer is the most vascularized one, containing various differentiated cells, such as astroglial cells that are able to express proangiogenic and prodifferentiating factors, almost absent in more inner layers. The cells of the peripheral layer show a low proliferative rate, as revealed by low levels of cell cycle marker, Ki67; in addition, they express very high levels of VEGF and low levels of HIF-1*α*. The intermediate layer, also called hypoxic layer, contains stem cells with high proliferative rate; in fact they tend to form neurospheres* in vitro* under hypoxic (1.5% O_2_) culture condition. These cells express high levels of HIF-1*α*, coexpressed with VEGF, and the highest levels of Glut1 and carbonic anhydrase IX (CAIX). The inner core is anoxic and stem cells in this area are mainly CD133^+^ according to the Nestin^+^ cells localization. Moreover, these cells show a strong expression of O6-methylguanine-DNA-methyltransferase (MGMT), due to higher presence of CD133^+^ cells, Glut1, and CAIX. Therefore, according to the tumor stem cell concentric model, the GBM mass may be represented as an anoxic core, with immature phenotype, surrounded by a hypoxic layer that shows an elevated proliferative rate; both these two layers are surrounded by a oxygenated and vascularized peripheral layer (Figure 1).

Thus, it emerges that the intratumoral hypoxia preserves a pool of tumor stem cells and the restricted oxygen conditions increase the GSCs fraction and promote acquisition of a stem-like state, as demonstrated by increase of expression of stem cell markers and reduction of the expression of differentiation markers. In this hypoxic conditions the proliferation rate and self-renewal potential of GSCs are also significantly increased as well as the expression of MGMT which is responsibel for GSCs resistance to alkylating chemotherapies (temozolomide) [152]. Therefore hypoxia drives directly, through its effect on tumor microenvironment and indirectly through the activation of hypoxia inducible factors, the tumor biology and the resistance to chemo- and radiotherapy.

### 3.6. Hypoxia Inducible Factors (HIFs) and Their Role in GSCs Tumorigenesis and Resistance

Recent reports suggest that hypoxia may have a pivotal role in tumor proliferation and malignant progression. Although it may affect negatively tumor cell growth, exposure to hypoxia induces malignant progression and aggressiveness, leading to increased resistance to therapy and a poor long-term prognosis. Cellular responses to hypoxia are commonly regulated by the hypoxia inducible factors (HIFs), a family of transcriptional factors which influence the transcription of several hypoxia inducible genes. HIF proteins are implicated in physiological and pathological adaptation to hypoxia, regulating the oxygen levels in cells and controlling the transcription of genes involved in critical aspects of cancer such as tumor progression, angiogenesis, drug and radioresistance, and cancer stem cells phenotype maintenance [153]. HIFs are members of the PAS (PER-ARNT- (aryl hydrocarbon receptor nuclear translocator-) SIM) family of basic helix-loop-helix (bHLH) transcription factors that bind to DNA as heterodimers, made of an oxygen-sensitive *α* subunit and a constitutively expressed *β* subunit, also known as ARNT. Three HIFs (HIF-1, HIF-2, and HIF-3) have been identified as key regulators of cellular transcriptional programs in response to oxygen levels [153, 154]. In fact, the degree of hypoxia differently influences the expression of HIF-1*α* and HIF-2*α*. In severe hypoxic conditions, both HIF-1*α* and HIF-2*α* resulted upregulated in GSCs while in mild hypoxia only HIF-1*α* is upregulated in glioma stem cells and in nonstem cells and in neural stem cells (NSCs), whereas HIF-2*α* is predominantly expressed only in GSCs [74]. HIFs are regulated by a series of oxygen-dependent modifications that are responsible for the regulatory cascade in hypoxic adaptation. In normoxic condition, HIF-1*α* protein stability is negatively affected by O_2_-dependent prolyl hydroxylation mediated by three HIF-specific prolyl hydroxylases (PDH1, PDH2, and PDH3), which induces the binding of the von Hippel-Lindau tumor suppressor protein (VHL), the subunit recognition of an E3 ubiquitin ligase that ubiquitytinates HIF-1*α*, targeting it for proteasomal degradation. O_2_-dependent asparagine-hydroxylation by factor inhibiting HIF-1 (FIH-1) prevents HIF-1*α* from interacting with coactivators [153, 154]. The cellular metabolic status can cooperate in the modulation of HIF-1*α* stability, as *α*-ketoglutarate, a Tricarboxylic Acid Cycle (TCA) intermediate, is a reaction substrate for prolyl hydroxylases (PHD) which can convert it to succinate and CO_2_ [153]. Therefore, under hypoxia, the activity of the prolyl-hydroxylases is inhibited and the affinity of VHL to HIF-1*α* is reduced, determining its rapid accumulation in O_2_-starved cells and the translocation into the nucleus where it forms dimers with HIF-1*β*, then recruiting coactivators p300 and CBP, binding to hypoxia response elements (HRE), containing the consensus binding site 5′-RCGTG-3′, within HIF target genes, and activating their transcription [153–157]. HIF-1*α* is the most ubiquitously expressed transcription factor and its increased level of has been positively correlated with tumor progression and poor prognosis in patients with brain cancers [153]. In contrast to the oxygen-dependent regulation of HIF-1*α*, growth factors stimulation induces its synthesis via a signal transduction pathway in which HER2, PI3K, the serine/threonine kinases AKT (protein kinase B), and FRAP (FKBP-rapamycin associated protein, also known as mammalian target for rapamycin mTOR) are involved. This pathway is negatively regulated by the tumor suppressor protein PTEN, which dephosphorylates the products of the PI3K reaction. Alterations of this pathway in cancer increase HIF activity [156]. In addition, mutations of VHL and of the genes encoding for succinate dehydrogenase (SDH) and fumarate hydratase (FH), that indirectly block ubiquitination by increasing the levels of TCA cycle intermediates, suppress prolyl hydroxylase activity and finally increase HIF activity under nonhypoxic conditions [156]. Targets of HIFs include members of stress-response gene families mediating acute and chronic hypoxic adaptations. These genes govern some crucial steps of tumorigenesis, including proliferation, metabolism, differentiation, angiogenesis, and metastasis [154]. Particularly the induction of HIF-1*α*, as a response to paracrine low oxygen pressure, allows the expression of genes promoting the reprogramming of tumor metabolism toward the glycolytic pathway, increasing glucose uptake, expression of glycolytic enzymes and lactate production, and regulating pyruvate metabolism in both hypoxic and normoxic (e.g., VHL deficient) cells [47, 156]. HIF can also control fatty acid and glycogen biosynthesis, activating the expression of the enzymes required to convert glucose to glycogen, including hexokinase (HK1 or HK2), phosphoglucomutase 1 (PGM1), UDP-glucose pyrophosphorylase (UGP2), glycogen synthase (GYS1), and glycogen branching enzyme (GBE1), as well as the gene encoding PPP1R3C, which activates GYS1 and inhibits liver-type glycogen phosphorylase (PYGL), the enzyme that breaks down glycogen [156]. In order to promote tumor growth and survival, the activation of HIF-1*α* enhances the expression of many proangiogenic factors, including VEGF, VEGF receptors FLT-1 and FLK-1, plasminogen activator inhibitor-1 (PAI-1), angiopoietins (ANG-1 and ANG-2), platelet-derived growth factor B (PDGF-B), and matrix metalloproteinases MMP-2 and MMP-9, that support tumor vascular remodeling and O_2_ and nutrients delivery [153, 154, 156, 157]. VEGF is detected at high levels in tumor areas and in proximity to necrotic area in GBM [153, 154]. HIF-1*α* was found to be highly expressed in GBM in particular in hypoxic cells forming pseudopalisades around regions of necrosis and in invading cells [158]. Most grade 3 astrocytomas showed strong staining for HIF-1*α*, as well [159]. HIF has been demonstrated to strongly promote metastatic processes in multiple tumors as has been reported to regulate fundamental factors mediating tumoral metastatic potential such as E-cadherin, lysyl oxidase (LOX), CXCR4, and stromal cell-derived factor-1 (SDF-1) [154]. It stimulates neovascularization and controls the invasion of GB cells through the recruitment of endothelial and pericyte progenitor cells [153]. In addition, hypoxia and HIF promote the undifferentiated state of GSCs, driving the initiation and progression of GB, and mediate the cancer insensitivity to radio- and chemotherapy, through the activation of Notch pathway [154]. Hypoxia strongly influences therapeutic resistance of tumor cells as on one hand it negatively impacts on the efficacy of some drugs and radiation that require oxygen to be maximally cytotoxic and on the other hand it promotes altered metabolism that reduces the drug cytotoxicity. Moreover, this condition enhanced genetic instability of tumor cells which may facilitate the more rapid development of drug resistance [160]. Several studies showed an increase in HIF-1 protein level following radiation. This is based on two possible mechanisms. Firstly, in hypoxia the number of stress granules, which are protein-mRNA complexes, increases, blocking the translation of HIF-1 mediated mRNAs into target proteins, whereas during radiation these complexes disaggregate leading to a burst of HIF-1 regulated proteins. Secondly, the radiation generates shortly excessive formation of free radicals species, which results in upregulation of HIF-1 activity [161]. Despite radiotherapy, up to 90% of all glioblastoma relapses in close proximity to the resection cavity, which is an area characterized by high expression of HIF-1 in tumor cells [162–165]. Another molecular mechanism showing the role of HIF-1 in drug resistance was the finding that HIF-1 is able to activate the multidrug resistance 1 (MDR1) gene in response to hypoxia. MDR1 encodes for the membrane-resident P-glycoprotein (P-gp) that belongs to a family of ATP-binding cassette (ABC) transporters. P-gp acts as drug efflux pump, decreasing the intracellular concentration of a range of chemotherapeutic drugs. MDR1 is a HIF-1 target gene and a contribution of HIF-1-mediated P-gp expression to hypoxia-induced drug resistance has been observed in numerous tumors including glioma. In addition, HIF-1 promotes the resistance to hypoxia initiated apoptosis through the stabilization of NF-*κ*B and the expression of antiapoptotic NF-*κ*B target genes. Therefore, HIF-1 functions as inhibitor of apoptosis since it also regulates many proapoptotic (e.g., BNIP3, NIX, and NOXA) as well as antiapoptotic factors (e.g., Bak, Bax, Bcl-xL, Bcl-2, Bid, Mcl-1, NF-*κ*B, p53, and survivin) [166]. In addition, the activation of HIF-1*α* enhances the recruitment of multiple BMDC populations via the CXCR4 pathway, which can induce local expression of VEGF, PlGF, VEGFR1, and CXCL12 [123].

## 4. ECM

On the basis of the finding previously described, it appears that many elements of the stem cells niche may contribute to the resistance to standard therapies. In particular, the low oxygen pressure and the consequent hypoxia strongly regulate components of the niche as well as the activation of prosurvival and proangiogenic pathways. However, GSCs not only interact with endothelial cells in perivascular regions but are also in contact with ECM, which is a complex system of macromolecules with specific physical, biochemical, and biomechanical properties and essential component of the niche. Abnormal ECM remodeling influences cancer progression, promoting cellular transformation and metastasis. The alteration of ECM affects cancer cells, acting on the behavior of stromal cells, including endothelial cells, immune cells, and fibroblasts that are responsible for ECM production. In addition, it facilitates tumor-associated angiogenesis and lymphangiogenesis resulting in the generation of a tumorigenic microenvironment [167]. The interaction between GSCs and specific ECM components within and around blood vessels is essential to understand how cell-ECM association produces effects on the response of tumor cells to radio- and chemotherapy. Several reports identified three main mechanisms by which ECM components might influence glioma stem cell survival and chemo- and radiosensitivity: (1) ECM proteins modulate responses to chemo- and radiotherapy; (2) ECM is a substratum for the activation of prosurvival integrin-mediated signaling cascades in tumor cells following radiation or chemotherapy; and (3) ECM creates a suitable niche for proliferation of cells that survive to irradiation or chemotherapy [168]. However, the heterogeneity of glioblastomas suggesting that the individual mechanisms proposed above may differentially affect the responses to treatments in the different patients should be considered. ECM proteins are the critical structural components of the perivascular niche and regulate normal stem cell and tumor proliferation and migration [77]. The overexpression of vascular basement components such as laminins has been associated with tumor grade and patient survival in gliomas; particularly laminin-8 is highly expressed in GBM and contributes to tumor invasion and regrowth after therapy [169]. The laminin receptor integrin *α*6*β*1 has been shown to regulate tumor cell survival, promoting endothelial cell growth in GBM [170]. Laminin is also important in the laminin-integrin relationship for GSCs maintenance, as it was revealed as fundamental component in adherent GSCs cultures [171, 172]. In addition, the component of the basement membrane, heparin sulphate, can bind basic Fibroblast Growth Factor (bFGF) [173], which stimulates growth [174] and inhibits radiation-induced apoptosis [175]. Integrins, transmembrane proteins, actively interact with the ECM, mediating many cell activities such as tissue morphogenesis, development, immune response, and cancer. In fact, these proteins allow the attachment of cells to ECM and the signals transduction across the cell membrane in response to the binding of ECM components such as laminin, fibronectin, vitronectin (VN), collagen, thrombospondin, and osteopontin [176–179]. The transmission of signals occurs through the formation of multimeric complexes known as focal adhesions with other signaling proteins such as focal adhesion kinase (FAK) [180]. Unbound integrins can transmit proapoptotic signals while complexed integrins activate growth and migratory pathways such as the MAPK, PI3K, NF-*κ*B, and Src pathways [76, 180, 181]. Integrins mediate the interaction between tumor cells and endothelial cells and between nontumoral stroma elements of the perivascular niche like pericyte and endothelial cells, regulating the function of the niche. Alteration in integrin expression is strongly associated with tumor malignant progression [76]. Integrin *α*6, the receptor for the ECM protein laminin, which forms heterodimers with integrin *β*1 or *β*4, is highly present in embryonic, hematopoeitic, and neural stem cells [182]. In the brain, laminins and integrin *α*6*β*1 regulate NCS growth [183] favoring the adhesion to the ventricular zone and their division [184]. It was observed in astrocytes [185, 186] and in glioma [187, 188], particularly in GBM cells with high levels of integrin *α*6, which were able to both self-renew and differentiate in CNS lineage, showing the stemness properties of GSCs [189]. In addition, integrin *α*3 is expressed in GBM cells, mainly in invading cells and in cells surrounding vessels* in vivo* and it regulates the invasive behavior of GSCs through the activation of ERK1/2 [190]. Integrin *β*1, highly expressed in perivascular niche, in GBM promotes invasion and along with CXCR4 can function in a signaling axis together regulating GSCs functions. Moreover, these proteins may modulate crucial stem cell pathways like the Wnt, SHH, and Notch pathways. Overexpression of integrins *α*v*β*3, *α*v*β*5, and *α*v*β*8 is correlated, respectively, with increased invasive and infiltrative phenotype of GBM and components of the niche like TGF-*β*1 and TGF-*β*2 can increase expression of *α*v*β*3 in tumor cells and increase their migratory activity [76]. Regarding cadherins, they mediate cell-cell interactions in a variety of biological processes such as tissue morphogenesis and tumor invasion and metastasis [191–193] and are critical for the maintenance of normal tissue structure including the neural stem cell niche. The cadherin induces adhesion-related signaling through its interaction, especially in CNS, with different regulators of fate and function including *β*-catenin, protein kinase C, cdc42, and Numb. Dynamic regulation of cadherin expression controls cell migration, fate, and function during normal development and oncogenesis. In the normal neural stem cell niche, N-cadherin expression is required to maintain the progenitor state. In GBM, the alteration of cadherin expression is associated with a change in tumor phenotype and growth; in fact, it was demonstrated that the antagonism of VEGF pathway generates a switch from angiogenic to infiltrative pattern of growth and alteration in integrin expression and also by a T to N cadherin switch. Similarly, Cadherin 11, a marker of mesenchymal subtype of GBM, enhances GBM cell migration and may be required for tumor growth* in vivo*. The expression of E-Cadherin in GBM patient specimens is associated with poor prognosis and a subset of E-Cadherin expressing CD133^+^ GSCs may have the capacity to transdifferentiate into endothelial cells. Cadherin expression is regulated by several transcription factors including FoxP2 and 4, Twist, and Snail and in cancer its expression is also controlled by cytokines like IL-8. Finally, interactions between cadherins and integrins have been recently observed in GSCs [76]. Of great interest is the role of ECM in tumor radioresistance and the enrichment of specific ECM components in the niche may have a protective role from external insults. In addition, it was shown that the radioresistance of glioma cell cultures is correlated with expression and activation of integrins *β*1 [194], *α*v*β*3, and *α*v*β*5 [195]. Moreover, the presence of* substrata* such as fibronectin and Matrigel increases survival in one glioma cell line evaluated after radiation treatment [196]. The expression of tenascin C stimulates tumor cell proliferation [197] and in glioma specimens correlates inversely with the degree of cell differentiation [198]. It was shown that radiotherapy increases tenascin C [199], and its overexpression is associated with a reduction of survival in GBM patients [200]. In addition, the interaction between glioma cells and ECM may inhibit apoptotic cell death. Interestingly, a report demonstrated that interaction between VN and integrin increases the expression of the antiapoptotic protein Bcl-2 or Bcl-X_L_ in glioma cells with a reduction in drug-induced programmed cell death and resultant chemoresistance [201]. VN and its receptors (the *α*v*β*3 and *α*v*β*5 integrins) are expressed at the tumor-brain interface in glioma cells and it is correlated with tumor grade [202–204].

## 5. GSCs Properties and Resistance to Traditional Therapies

Conventional therapies target the tumor bulk but have no efficacy toward the GSCs compartment, as GSCs display by themselves specific biological features and different mechanisms, which are implicated in their survival and are responsible for their resistance to treatments:

1. Drug efflux system consisting of ATP-binding cassette (ABC) membrane transport proteins such as ABCG2 that use the energy derived from ATP hydrolysis to actively pump compounds like drugs out of the cytoplasm of tumor cells.
2. Cell cycle regulation and DNA repair systems. BTSCs can activate ATM (ataxia telangiectasia mutated) and ATR (ataxia telangiectasia and Rad3 related) that firstly respond to genotoxic stress and DNA damage activating signal transduction pathways mediated by the effector kinases Chk1, Chk2, and Rad17. The effector or checkpoint kinases work activating p53 and inactivating cyclin-dependent kinases, to halt cell cycle progression and allow DNA repair. The repair enzyme MGMT functions by removing methyl groups from O6-guanine after treatment with temozolomide and allows the cell to continue the replication.
3. Antiapoptotic mechanisms [205].
4. Activation of specific pathways such as Wnt/*β* catenin, SHH, and Notch implicated in tumor radio- and chemoresistance [205, 206].

## 6. Conclusions

We have reported and discussed many studies demonstrating that GSCs reside in particular tumor niches that are necessary to support their behavior. A hypoxic microenvironment has been finally demonstrated to play a crucial role in controlling GSC molecular and phenotypic profile and in promoting the recruitment of vascular and stromal cells in order to sustain tumor growth. Recent advances in the field allow researchers to generate models able to simulate, at least in part, the extreme heterogeneity found within GBM tumors. These models try to account for the presence of GSCs and more differentiated cells, the influence of different microenvironments enclosed within the mass, heterotypic interactions between GBM and stromal cells, and genetic aberrations. Understanding the mechanism of action of the microenvironmental signals and the interplay between different cell types within the tumor mass open new questions on how GSCs modulate GBM aggressiveness and response to gold standard therapies. The definition of these tumor features will allow setup of innovative multimodal therapies able to target GBM cells at multiple levels. Drug-resistant CSC-like cells, such as GCSs, which very likely underlie disease relapse, are a key obstacle for curing cancer. Overcoming this obstacle in GM is an important objective, even if difficult to achieve, because our understanding of GSCs and their microenvironment-dependent pathways of drug resistance is still limited. Knowledge gaps remain in our comprehension of the cellular signal transduction cascades that govern the maintenance of GSCs* in vivo*. Moreover, it should be taken into account, as reported above, that many possible ways of intervention, that is, antibody anti-VEGF, may increase GSCs aggressiveness, thus resulting in worse outcome. The same may happen when only the gold standard therapies are administered, that may result also in an increase of GSCs proliferation. In recent years, molecularly targeted drugs have joined conventional chemo- and radiotherapies for the management of several cancers and have become the first-line treatments for tumors lacking efficacious therapeutic options, such as the approval of bevacizumab for recurrent GBM. Benefits of targeted therapy in terms of overall survival are modest; however, in GBM, whose median survival is approximately 15 months, even an improvement of progression-free survival could be encouraging. This may be due to the unavailability at presence of a multimodal approach against all the actors playing a role in GSCs maintenance, proliferation, and migration.

Taken together all the factors concurring in the formation of the niche, mainly dependent on pO_2_, suggest the importance to study on one hand the biology of GSCs that confers itself resistance to therapies and on the other hand the tight relationship between GSCs and the microenvironment of the niche, highlighting the variety of mechanisms that might contribute to the chemo- and radioresistance. In order to identify the specific therapeutic target, it is, therefore, crucial to further investigate the mechanisms of radio- and chemoresistance promoted by the components/factors of the niche. This would probably allow formulating new adjuvant therapies rendering more efficiently the gold standard therapies for this neoplasm.

## Figures and Tables

**Table 1:** Models of gliomagenesis. Summary of genetically engineered murine models and lentiviral systems which are targeted specific CNS cell types to reproduce the genetic alterations of GBM, the gliomagenic process, and to detect the tumor cell of origin.

Model types and mutations in targeted CNS cells	Cell of origin of glioma
Holland et al., 2000 [21] Ras/Akt mutations in neural progenitors (Nestin^+^) and in differentiated astrocytes (GFAP+)	Nestin expressing cells: NSCs and progenitor cells

Alcantara Llaguno et al., 2009 [22] p53, PTEN, and NF1 knockout mice	Nestin expressing cells: NSCs or progenitor cells in SVZ

Wang et al., 2009 [23] Mutated p53 model	Nestin/oligo2-positive cell population

Lindberg et al., 2009 [24] Liu et al., 2011 [25] P53 and NF1 knockout mice	OPCs

Koso et al., 2012 [26] A transposon-mediated mutagenesis Approach in isolated mouse NSCs	Astroglia mutagenized; NSCs most sensitive to oncogenic transformation after differentiation to the astrogial lineage

Dai et al., 2001 [27] PDGFR activation via RCAS-tVA	Neural progenitors in the SVZ

Marumoto et al., 2009 [28] Lentiviral delivery of Kras/Akt oncogenes	Neural progenitors in the SVZ

Zheng et al., 2008 [29] Jacques et al., 2010 [30] PTEN/p53 inactivation	Neural progenitors in the SVZ

Bachoo et al., 2002 [31] Ink4a-ARF knockout	NSCs and astrocytes

Bruggeman et al., 2007 [32] Bmi knockout	NSCs and astrocytes

Uhrbom et al., 2002, 2005 [33, 34] Combined Ink4a-ARF knockout and Kras activation	Neural progenitors and astrocytes

NCSs: neural stem cells, OPCs: oligodendrocyte progenitor cells (modified from Modrek et al., 2014 [38]).
